# Electrostatic Interaction-Induced Assembly of Colloidal Photonic Crystals on Polyester Yarns Applied for Structurally Colored Fabrics with High Breathability and Colorfastness

**DOI:** 10.3390/polym18192395

**Published:** 2026-09-30

**Authors:** Xiaohui Wang, Yalan Zhang, Xinyang Li, Jianjun Guo, Wanyang Sun, Yichen Li, Guojin Liu, Yi Huang, Minghua Wu, Jianzhong Shao

**Affiliations:** 1 Engineering Research Centre for Eco-Dyeing and Finishing of Textiles, Ministry of Education, Zhejiang Sci-Tech University, Hangzhou 310018, China; 2 Rongsheng Petrochemical Co., Ltd., Hangzhou 310018, China; 3 School of Textile and Clothing, Soochow University, Suzhou 215127, China; 4 Hangzhou PhocryColor Sci-Tech Co., Ltd., Hangzhou 311200, China

**Keywords:** photonic crystals, structural coloration, yarn, breathability, electrostatic interaction-induced assembly

## Abstract

Structurally colored technology based on photonic crystals (PCs) shows significant potential for applications in ecological coloration, fashion textiles, and smart textiles. However, structurally colored fabrics with photonic crystals (SCFa-PCs) prepared by the coating method usually have poor air permeability. Preparing structurally colored yarns with photonic crystals (SCY-PCs) and subsequently weaving them into fabrics is an effective approach to addressing air-permeability limitations. Nevertheless, constructing PC structures with both high structural stability and high color saturation on polyester yarns remains a significant hurdle due to their complex surface morphology and hydrophobicity. Herein, ionic surfactants are employed as surface-modifying agents to enhance the affinity of polyester yarns for polystyrene @ poly(methyl acrylate-butyl acrylate) [PS@P(MMA-BA)] colloid nanosphere assembly liquid, enhancing the assembly efficiency and uniformity of PS@P(MMA-BA) PCs. The reflection curve of the prepared SCY-PCs has a narrow and high peak, which proves the high brightness and high saturation of the structural color. In the fabricated SCY-PCs, due to the self-adhesive property of PS@P(MMA-BA) nanospheres, the PC structure on the fiber surface possesses excellent structural stability under various conditions such as rubbing and washing. Subsequent textile processing, including weaving and embroidery, produces structurally colored fabrics with high breathability, outstanding color fastness, and high color saturation. The visible color of the SCY-PCs can be precisely controlled over the spectral range of 400–700 nm by adjusting the diameter of the nanospheres. This innovative approach addresses the air-permeability limitations of bionic SCFa-PCs and reveals promising capability for the broader application of structurally colored technologies in textile manufacturing.

## 1. Introduction

Photonic crystals (PCs) [1,2,3,4] are a class of crystalline materials formed through the spatially periodic arrangement of two or more substances with different dielectric constants. In nature, opals, peacock feathers, and chameleon skin are well-known examples of typical PC structures [5]. Unlike traditional dyes and pigments, which often suffer from photobleaching and environmental degradation, PCs not only deliver structural colors with high brightness, saturation, and tunability but also possess outstanding lightfastness and long-term weather stability. Unlike conventional dyeing processes that depend predominantly on chemical dyes, pigments, and auxiliary additives, structural coloration stemming from PCs significantly reduces wastewater generation and energy consumption. These advantages align with the principles of green manufacturing in the modern textile industry. Thus, PC-based structural coloration technologies show significant application potential and promising development prospects in the textile-related field [6,7,8,9].

Current applications of PC materials in the field of textiles primarily include structurally colored fibers [10,11], structurally colored yarns [12,13], and structurally colored fabrics [14,15,16,17]. Among these, the main fabrication methods for structurally colored fibers based on photonic crystals (SCFi-PCs) include electrospinning [18,19,20], microfluidic techniques [21,22,23], and extrusion processes [24]. However, SCFi-PCs prepared by these methods often suffer from a compromise between color saturation and mechanical performance. In contrast, structurally colored fabrics embedding photonic crystals (SCFa-PCs) have been extensively studied. Key challenges, including structural stability [25,26,27], large-area assembly uniformity, and rapid fabrication efficiency [28,29], have been substantially addressed. Shao et al. [30] employed liquid photonic crystals (LPCs) as an assembly precursor to enable rapid, large-area fabrication of SCFa-PCs through shear-induced assembly, with structural stability enhanced by co-assembled soft particles. Nevertheless, the extent of stability improvement remained limited. To further improve structural robustness, Shao et al. [31] designed a non-close-packed PC structure on fabric surfaces using hard-core/soft-shell nanospheres as building blocks. The resulting SCFa-PCs exhibited excellent structural stability and high color saturation. However, the PC structure detrimentally affected permeability, thereby reducing fabric comfort. Ge et al. [32] fabricated breathable SCFa-PCs with high structural stability via a spray-based method. Despite this advancement, the bulk production of monodisperse SiO_2_ nanospheres poses ongoing difficulties, which limit practical application. Compared with SCFi-PCs and SCFa-PCs, SCY-PCs have several advantages. First, yarns provide a larger effective assembly area than individual fibers. Second, yarns allow more flexible and diverse processing routes than fabrics. Nevertheless, the preparation of SCY-PCs has many challenges. Achieving PC assemblies on yarn surfaces that concurrently demonstrate high structural stability and high color saturation is more difficult than on fabric substrates. Fabrics present relatively flat two-dimensional surfaces, allowing uniform nanosphere packing and ordered assembly. In contrast, yarns are three-dimensional substrates with intrinsic curvature, and twisted yarns contain numerous surface grooves, both of which hinder the regular assembly of PCs.

Herein, polystyrene @ poly(methyl acrylate-butyl acrylate) [PS@P(MMA-BA)] nanospheres are employed as the building blocks of PCs, and ionic surfactants modify polyester yarns. Driven by electrostatic induction, the colloidal nanospheres rapidly assemble and crystallize on the yarn surface, generating bright structural colors and enabling the efficient fabrication of structurally colored yarn based on photonic crystals (SCY-PCs). Subsequently, through weaving, knitting, and embroidery, the SCY-PCs are further processed into structural color textiles. The non-close-packed PCs assembled by PS@P(MMA-BA) nanospheres possess excellent flexibility and structural stability, making them compatible with flexible polyester yarns. This strategy efficiently reconciles color saturation with structural stability in SCY-PCs and facilitates rapid, continuous PC assembly on polyester yarns, as well as the large-scale preparation of breathable SCFa-PCs, demonstrating considerable promise for practical applications.

## 2. Experimental Section

### 2.1. Materials

Styrene (St), divinylbenzene (DVB), methyl methacrylate (MMA), butyl acrylate (BA), allyl methacrylate (ALMA), sodium dodecyl sulfate (SDS), potassium persulfate (KPS), sodium hydroxide (NaOH), and cetyltrimethyl ammonium bromide (CTAB) were purchased from Aladdin Reagent Co., Ltd. (Shanghai, China) at analytical grade. Nano carbon black (Emperor 1800) was sourced from Shanghai Cabot Chemical Co., Ltd. (Shanghai, China). Deionized water with a conductivity of 18 MΩ cm^−1^ was produced in-house. Polyester yarns (150 D) were bought from a local commercial supplier.

### 2.2. Preparation of PS@P(MMA-BA) Nanospheres

PS@P(MMA-BA) nanospheres were synthesized via a two-step emulsion polymerization in a four-necked flask equipped with a nitrogen bubbler, reflux condenser, and paddle-type agitator [31]. Initially, 0.15 g of SDS, 10 g of St, and 1 g of DVB were added to 250 mL of water and emulsified for 10 min at 380 rpm. Subsequently, 0.65 g of KPS dissolved in 50 mL of water was introduced into the reaction system to initiate polymerization for 2 h. Then, 30 g of St and 4 g of DVB were added to the reaction mixture. After 60 min, 20 g of MMA, 35 g of BA, and 0.16 g of ALMA were slowly added. The reaction proceeded at 80 °C for 1 h.

### 2.3. Preparation of Assembly Working Liquid

The PS@P(MMA-BA) nanosphere dispersion was mixed with an appropriate amount of aliphatic polyoxyethylene ether and concentrated by rotary evaporation to obtain an LPC with bright structural colors. Glycerol (3 wt%) and nano-carbon black (0.06 wt%) were added to the LPC, with the weight percentages calculated relative to the weight of the LPC. The mixture was magnetically stirred at 20 r/s for 10 min to ensure uniformity, yielding the assembly working liquid [31].

### 2.4. Modification of Polyester (PET) Yarns

Polyester yarns were submerged in a 50 g/L SDS aqueous solution for 1 min, then removed and dried in an 80 °C oven.

### 2.5. Preparation of SCY-PCs

The modified polyester yarns were immersed in the assembly working liquid for 5 s, then removed, and the excess liquid was removed from the yarn surface using a sponge. The SCY-PCs were prepared using a two-stage assembly method. The assembly process was carried out in two sequential steps. The pre-assembly step was performed on a pre-assembly roll at 90 °C for 2 min, followed by a secondary assembly treatment in an assembly box at 130 °C for 2 min. After complete evaporation of the water, yarns with structural colors assembled from PS@P(MMA-BA) nanospheres were obtained. The SCY-PCs with different structural colors could be obtained by using PS@P(MMA-BA) nanospheres with different particle sizes.

### 2.6. Testing and Characterization

#### 2.6.1. Surface Morphology

The arrangement of nanospheres and PCs was observed using a field emission scanning microscope (FESEM, ULTRA55, Carl Zeiss, Oberkochen, Germany).

#### 2.6.2. Optical Properties

The structural color of the LPC and PC was examined with an optical microscope (KH-7700, Hirox-USA, Inc., Hackensack, NJ, USA) and a digital camera (EOS 600D, Canon Inc., Tokyo, Japan).

Reflectance spectra of the LPC and structurally colored PET yarns were analyzed using fiber optic spectroscopy (Maya 2000, Ocean Optics, Dunedin, FL, USA). Data were acquired with an integration time of 8 s and 200 integrations, and each spectrum was averaged over 3 accumulations.

#### 2.6.3. Mechanical Properties

The mechanical properties of the SCY-PCs were tested using a universal material testing machine (Instron 3345, Instron, Norwood, MA, USA). The test environment and parameter settings were as follows: temperature 20 ± 2 °C, relative humidity 65 ± 3%, clamp distance 500 mm, and stretching speed 500 mm/min.

#### 2.6.4. Moisture Permeability

The moisture permeability of the fabric was tested using a fabric moisture permeability tester (FX3150, TEXTEST, Schwerzenbach, Switzerland). The sample was allowed to equilibrate for 60 min at a temperature of 38 ± 2 °C and a relative humidity of 50 ± 2%. Then, the test program was initiated, and the results were averaged after three tests.

#### 2.6.5. Air Permeability

The air permeability was tested using a permeability tester (KES-F8, KATO TECH Co., Ltd., Kyoto, Japan). The airflow passed through the fabric and then returned to the atmosphere. The inhalation and exhalation phases each lasted for 5 s. The air resistance of the sample was measured by a semiconductor differential pressure gauge. In this work, air resistance (AR) was used as an indicator of the air permeability of the fabric. AR is inversely related to air permeability: a higher AR value corresponds to lower air permeability, whereas a lower AR value indicates better air permeability.

#### 2.6.6. Structural Stability

The fabric woven with SCY-PCs was first immersed in an acidic solution (pH = 3) and an alkaline solution (pH = 11) for 5 min each, then taken out and dried. Subsequently, it was rubbed with sandpaper and toothbrushes 50 times. Finally, it was washed with a 5 g/L soap powder aqueous solution at 60 °C for 30 min. The fabric that completed the above process was then dried, and the reflectance curve of structural color was tested and compared with the original sample.

## 3. Results and Discussion

### 3.1. Optical Properties of PS@P(MMA-BA) LPCs

**Figure 1:**
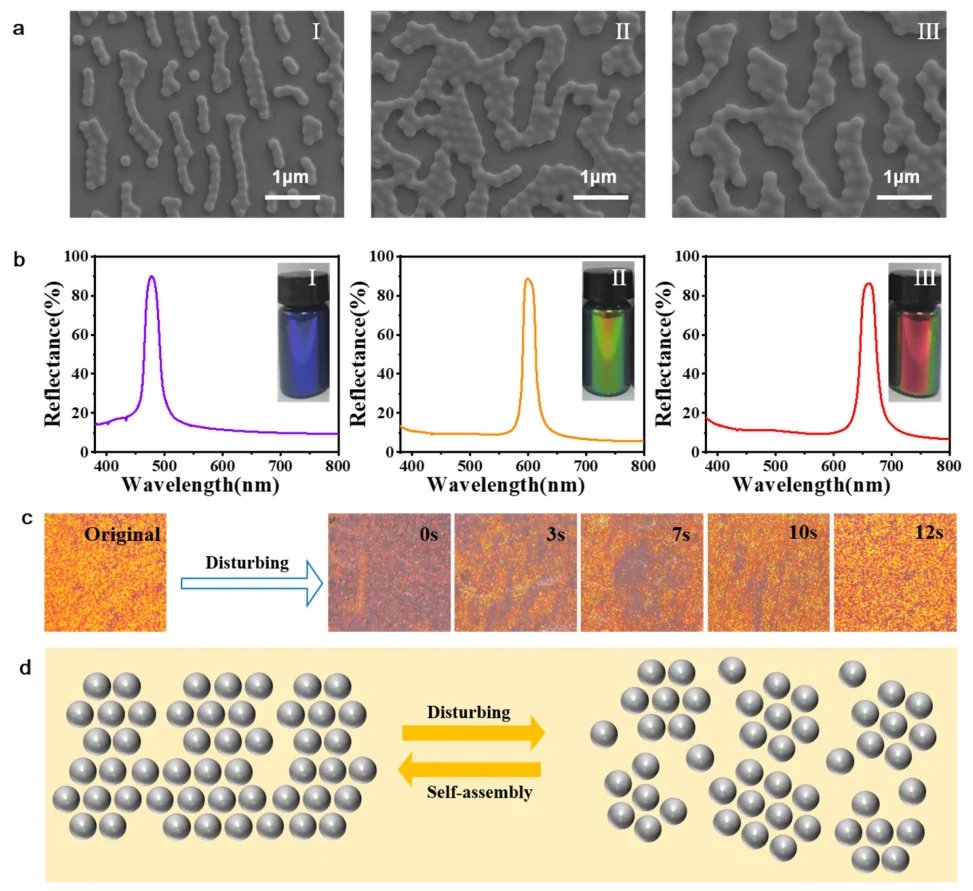
PS@P(MMA-BA) nanospheres and LPCs: (**a**) SEM images of PS@P(MMA-BA) nanospheres featuring different particle sizes ((**I**) 198 nm, (**II**) 255 nm, and (**III**) 302 nm); (**b**) reflectance spectra and corresponding digital photographs of the LPCs; (**c**) optical microscopy images of the LPCs during external disturbance; (**d**) schematic diagram illustrating dynamic recovery of the LPCs.

Figure 1 presents the SEM images of PS@P(MMA-BA) colloidal nanospheres with a hard-core/soft-shell, together with the corresponding optical properties of the LPCs. As shown in Figure 1a and Figure S1, PS@P(MMA-BA) nanospheres with different particle sizes exhibit good sphericity and uniform morphology. Owing to the presence of the soft shell, the PS@P(MMA-BA) nanospheres reveal enhanced deformability and superior self-adhesion properties [31]. Figure 1b shows the reflectance spectra and corresponding digital photographs of PS@P(MMA-BA) LPCs with different structural colors. The maximum reflection wavelength ($\lambda_{max}$) corresponds to the photonic bandgap position, i.e., the hue, while the peak height and peak width reflect the brightness and saturation of the structural color. Reflectance curves combined with optical microscope images are therefore sufficient to characterize the color properties of the PCs. As shown in Figure 1b, the reflectance spectra display high intensity and narrow reflection peaks, indicating remarkable optical properties of the LPCs. The maximum reflection wavelength of the reflectance curve corresponds to the structural color of the LPCs. In addition, with increasing particle size, the structural color of the LPCs exhibits a red shift, which is consistent with Bragg’s diffraction equation [30].

$$\lambda_{\max}=2\;D{\left(n_{eff}^{2}-\sin^{2}\theta \right)}^{1/2}$$

where $\lambda_{max}$ is the central wavelength of reflected light corresponding to the photonic bandgap (PBG) position, *D* denotes the interplanar spacing of the (111) crystal planes, *n_eff_* represents the effective refractive index of the PC, and θ is the angle between the incident light and the surface normal of the sample (equivalent to the viewing angle). When the effective refractive index of the PC is held constant, $\lambda_{max}$ can be tuned by changing the particle size of the nanospheres. Specifically, increasing the particle size of the PS@P(MMA-BA) nanospheres leads to a larger $\lambda_{max}$ value, causing the structural color to red-shift.

The dynamic reversible recovery behavior of PS@P(MMA-BA) LPCs is illustrated in Figure 1c. In the absence of external perturbation (initial state), the LPC exhibits a large area of crystal clusters with structural colors. After an external disturbance (0 s), the color saturation and brightness of the structural color of the crystal clusters decrease significantly. Notably, the structural color recovers to its initial state within 12 s after elimination of the disturbance. Figure 1d schematically depicts the dynamic recovery diagram of the LPC. In the static state, PS@P(MMA-BA) nanospheres arrange in a highly ordered face-centered cubic (FCC) structure. External disturbance destroys this regular arrangement, converting the ordered FCC structure into a disordered amorphous structure. Once the external force is removed, the nanospheres rapidly reassemble into the thermodynamically favored FCC structure under the combined effects of electrostatic repulsion and van der Waals attraction, thereby restoring the bright structural colors.

### 3.2. Preparation of SCY-PCs and Electrostatic-Induced Assembly Mechanism

**Figure 2:**
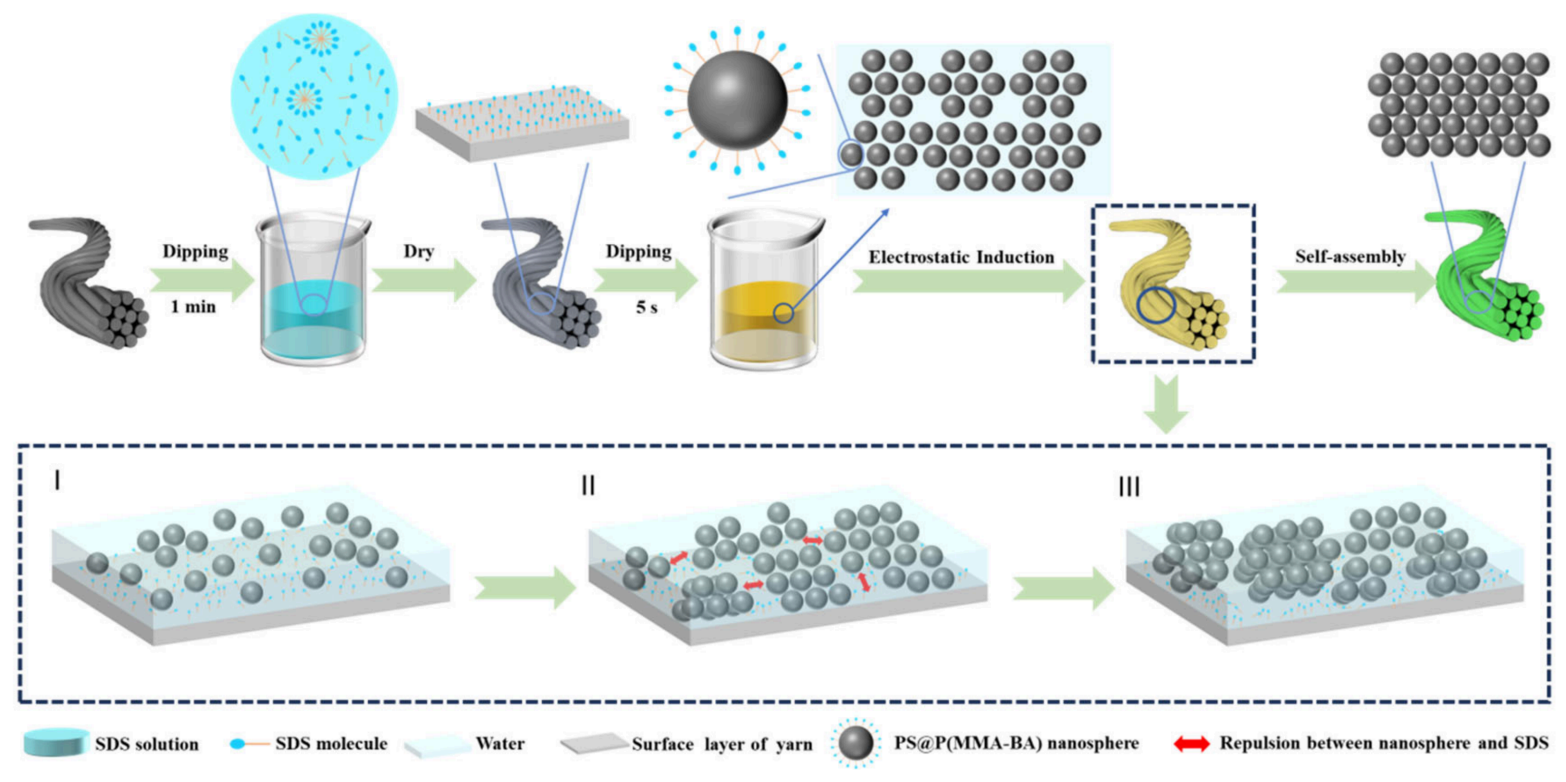
Schematic illustration of the preparation process and the electrostatically induced self-assembly mechanism of SCY-PCs.

Due to the inherent hydrophobicity of polyester fibers and the compact structure of twisted polyester yarns, the aqueous assembly liquid does not readily spread uniformly on the yarn surface. Therefore, ionic surfactants were used to modify polyester yarns, significantly improving their surface wettability and enhancing their ability to receive the aqueous assembly liquid. As shown in Figure 2, polyester yarns were immersed in a 50 g/L SDS aqueous solution, followed by drying at 80 °C after adsorption of SDS molecules onto their surface. Because the glass transition temperature of polyester fibers is higher than 80 °C, the SDS molecules could not diffuse into the fiber interior and were retained only on the surface of the polyester yarns and fibers. Subsequently, the yarns were placed in the assembly liquid for 5 s and then rapidly withdrawn, and excess assembly liquid on the surface was wiped off (Figure S2). This step simulates the “one dip and one squeeze” process used in continuous production. The wiping step serves to control the liquid uptake and ensure uniform liquid coverage on the yarn surface. The polyester yarns containing an appropriate amount of assembly liquid were then heated to induce assembly, yielding the formation of SCY-PCs with bright colors. Based on our previous research [33] and the results presented in this paper, Figure 2 schematically illustrates the electrostatic-induced self-assembly mechanism (steps I–III in the dashed box).

Step I (Figure 2I): The colloidal nanospheres adsorbed on the polyester yarns transition from an LPC state to a monodisperse state.

Step II (Figure 2II): The assembly liquid forms a thin water film on the yarn surface, which redissolves and ionizes the SDS molecules previously adsorbed on the yarns. The negatively charged SDS molecules generate electrostatic repulsion toward the negatively charged colloidal nanospheres adsorbed on the yarns, driving the nanospheres to assemble in the gradually thinning aqueous phase space.

Step III (Figure 2III): When the interparticle distance decreases to a critical level, crystallization occurs, and the colloidal nanospheres reassemble into an LPC with a quasi-crystalline state on the yarn surface, producing bright structural colors. As water evaporates, further self-assembly takes place, and the LPCs gradually transform into solid PCs. During this process, the interplanar spacing decreases, leading to a blue shift of the structural color, such as the green color shown in Figure 2.

To further elucidate the role of ionic surfactants in the self-assembly of nanospheres more intuitively, the polyester yarn substrate was replaced with a polyester fabric substrate, which provides a larger surface for visualizing electrostatic induction effects. As shown in Figure S3, the PCs assembled on fabric surfaces modified with anionic or cationic surfactants display irregular patterns, whereas those assembled on fabric surfaces modified with nonionic surfactants do not display such features. This difference suggests that electrostatic interactions between ionic surfactants and charged colloidal nanospheres play a key role. Two aspects are worth noting. First, the irregular patterns observed on the fabric may also be interpreted as uneven coloration, which is generally considered undesirable. However, for polyester yarns, owing to their small diameter and inherent curvature, local regions of relatively high-concentration crystallization points and low-concentration non-crystallization points induced by electrostatic interactions at an appropriate surfactant concentration cannot be resolved by the human eye. These regions are similar to high-density “pixel” points, resulting in a macroscopically uniform appearance. Thus, the brightness and saturation of the structural color of polyester yarns are significantly enhanced through electrostatically induced assembly. Second, from a macroscopic perspective, polyester yarns modified with anionic surfactants and those modified with cationic surfactants exhibit similar electrostatic induction assembly effects. However, from a microscopic perspective, the underlying mechanisms are different. After modification with an anionic surfactant, the substrate becomes negatively charged. Due to the electrostatic repulsion between the negatively charged substrates and the negatively charged colloidal nanospheres, the nanospheres are repelled back into the aqueous phase. As water continuously evaporates during self-assembly, the liquid film thins, and the nanospheres re-entering the aqueous phase become progressively closer, promoting faster formation of a thermodynamically stable PC structure. In contrast, substrates modified by cationic surfactants attract negatively charged colloidal nanospheres through electrostatic attraction between opposing charges, causing local accumulation and crystallization on the modified substrate.

**Figure 3:**
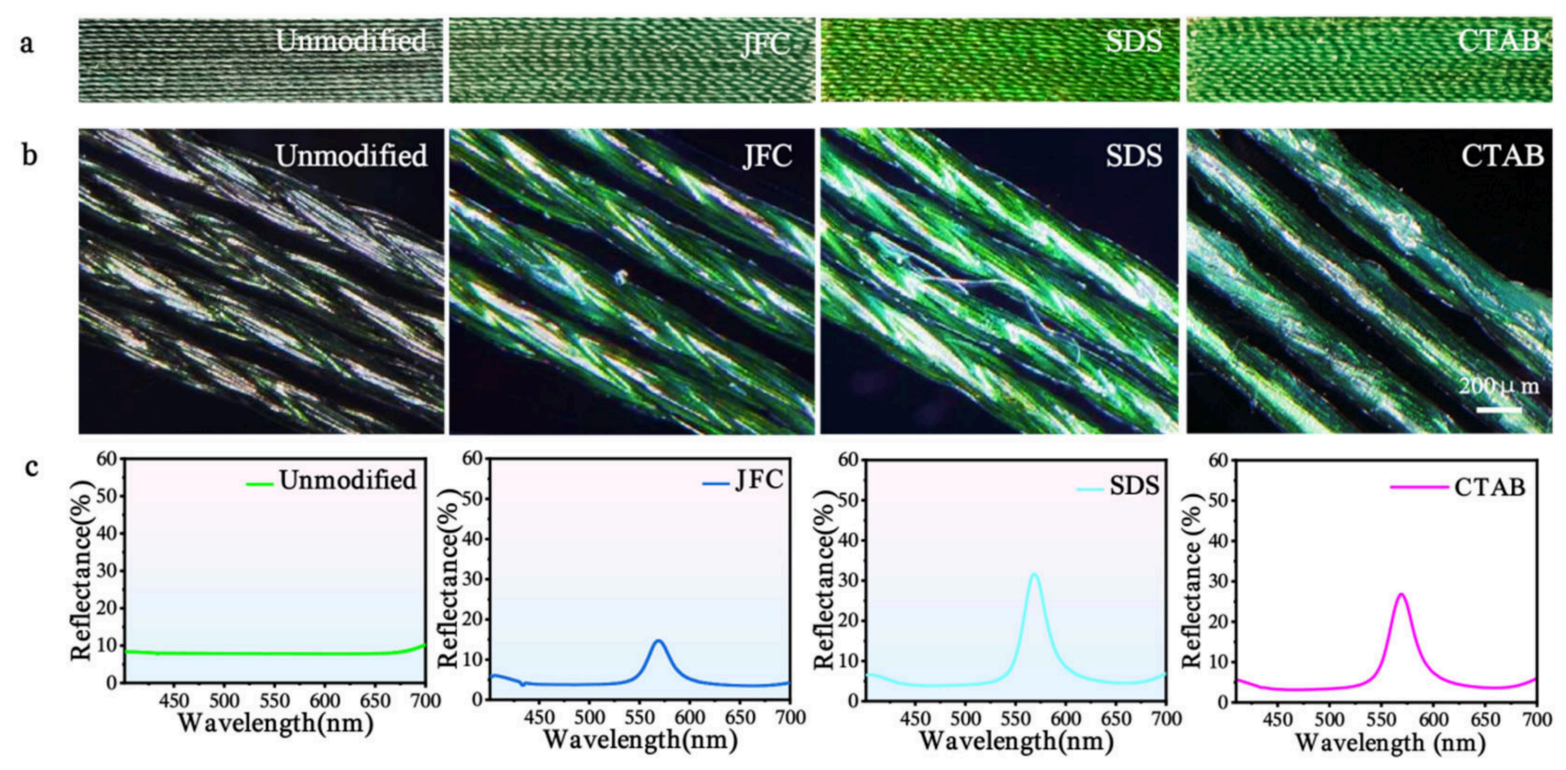
Optical properties of PCs assembled on the surfaces of polyester yarns under different modification conditions: (**a**) digital images, (**b**) optical microscope images, and (**c**) reflectance spectra.

Figure 3 shows the optical properties of PCs assembled on polyester yarns under different surface modification conditions. PCs assembled on polyester yarns modified with anionic or cationic surfactants exhibit significantly enhanced optical properties compared to those formed on unmodified and nonionic surfactant-modified polyester yarns. This result can be explained based on Figure 2. When unmodified polyester yarns are immersed in the assembly liquid, the poor wettability limits liquid uptake, resulting in the absorption of only a small amount of assembly liquid. Consequently, the colloidal nanospheres do not have sufficient time to assemble, resulting in a poor coloration effect. Modification with non-ionic surfactants improves liquid absorption and increases the number of nanospheres on the yarn, thereby enhancing structural color. However, this effect remains inferior to that achieved with ionic surfactants. After modification with the anionic surfactant SDS, not only is liquid uptake enhanced, but the assembly of colloidal nanospheres is also accelerated by electrostatic induction, producing bright and vivid structural colors with higher and narrower reflection peaks. Polyester yarns modified with cationic surfactants demonstrate structural coloration similar to that of yarns modified with anionic surfactants. Based on the optical performance of SCY-PCs, as well as the cost and availability of the modifiers, a 50 g/L SDS solution and 40 wt% nanosphere LPCs were selected as the optimal modification and assembly conditions (Figures S4–S6).

**Figure 4:**
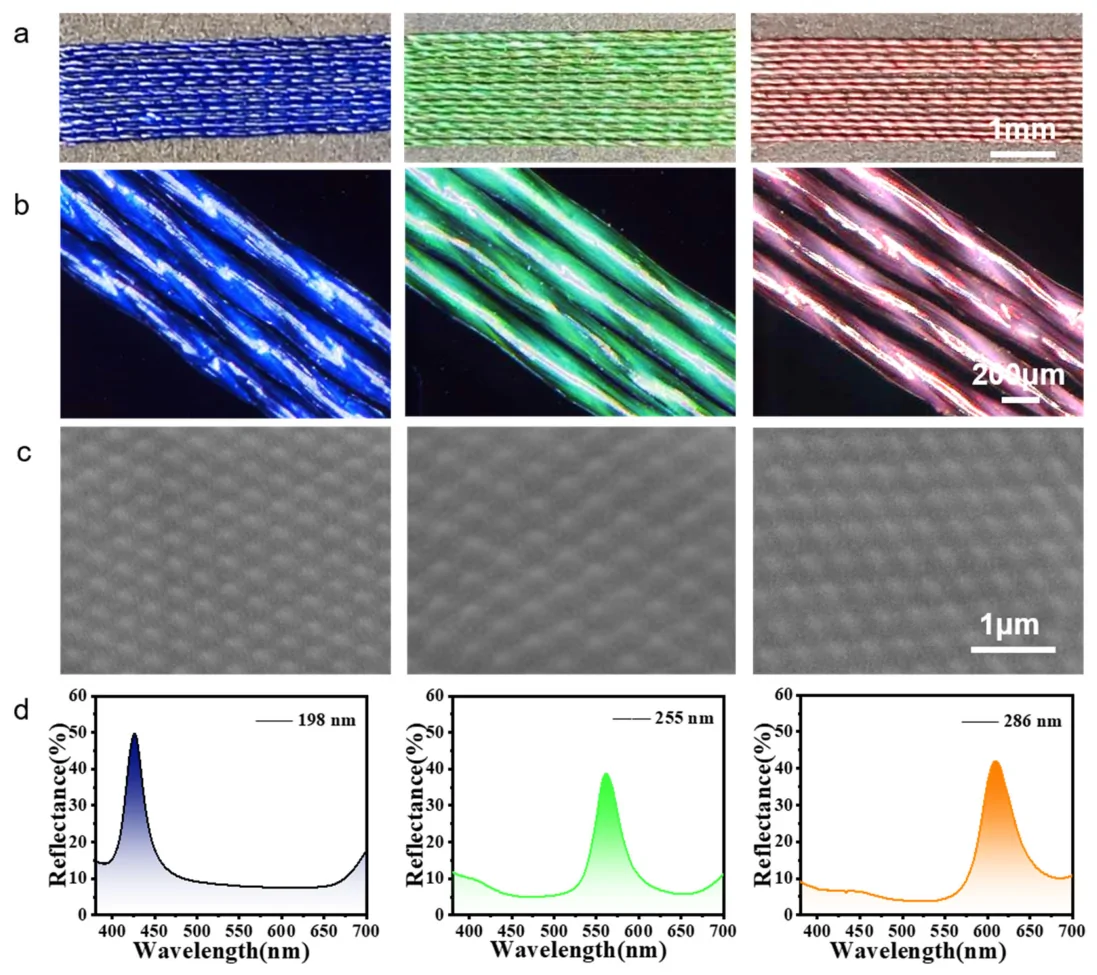
Optical properties of SCY-PCs: (**a**) digital images, (**b**) optical microscope images, (**c**) SEM images, and (**d**) reflectance spectra. The particle sizes of the nanospheres from left to right are 198 nm, 255 nm, and 286 nm, respectively.

Figure 4 shows the SCY-PCs prepared from PS@P(MMA-BA) nanospheres with different particle sizes. As shown in Figure 4a,b, the SCY-PCs possess high brightness and saturation. The variation in structural color follows the Bragg diffraction formula. As the particle size of the nanospheres increases, the structural color shifts towards red. The corresponding SEM image (Figure 4c) reveals a highly ordered arrangement of nanospheres with a typical face-centered cubic stacking structure. No air phase among the nanospheres; instead, they are fused through their soft shell layers. Figure 4d presents the reflectance spectra of three different colored SCY-PCs. The reflectance spectra of the SCY-PCs display high and narrow reflection peaks, which indicates that the structural color possesses high brightness and saturation. Moreover, the maximum reflection wavelengths of the three reflectance curves are 426 nm, 561 nm, and 609 nm, respectively, matching the displayed colors.

### 3.3. Continuous Preparation and Application of SCY-PCs

**Figure 5:**
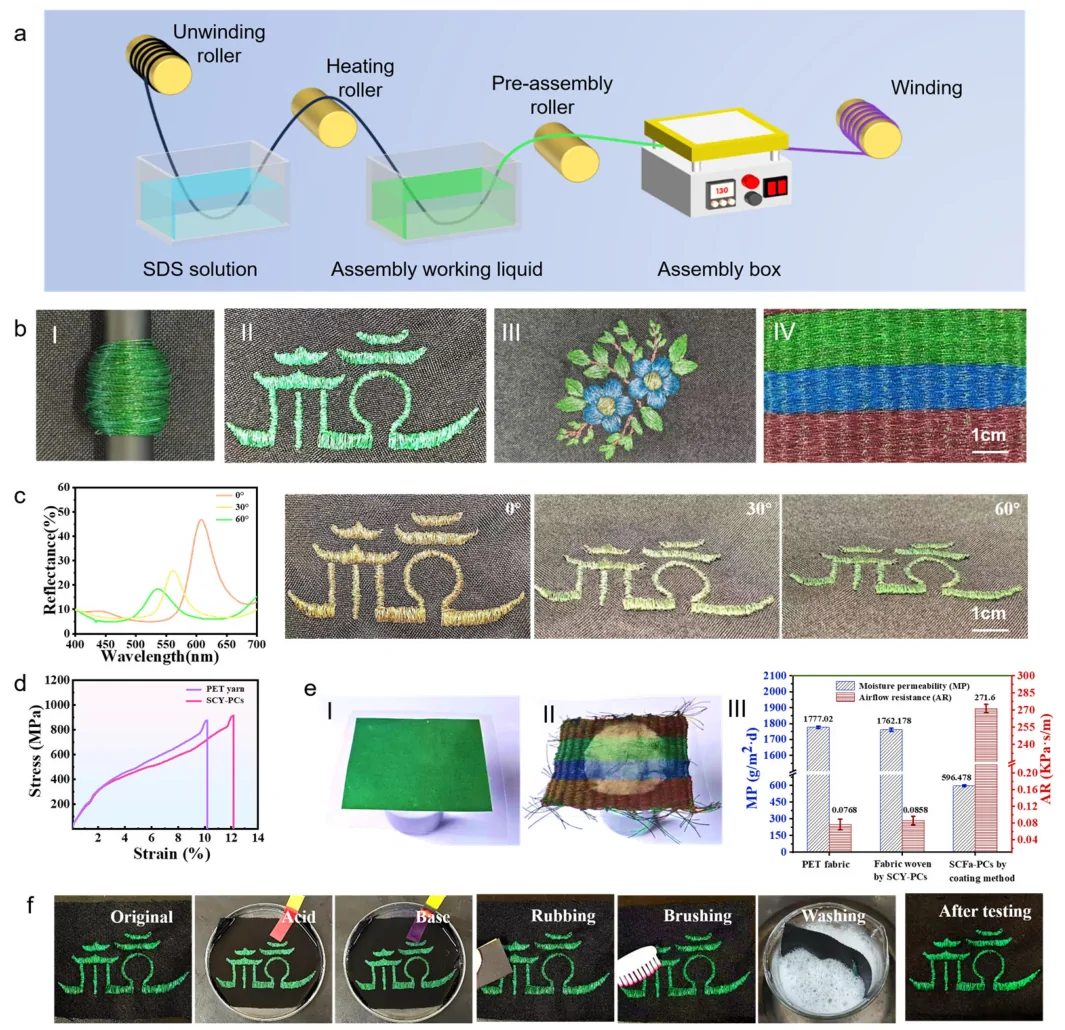
Continuous preparation and properties of SCY-PCs: (**a**) schematic illustration of the continuous preparation process; (**b**) digital images of SCY-PCs (**I**) and fabrics woven from the SCY-PCs (**II**–**IV**); (**c**) reflectance spectra and digital images of SCFa-PCs (red yarn region in panel (**b**(**IV**))) measured at different angles; (**d**) stress–strain curve of PET yarn and SCY-PCs; (**e**) moisture permeability and breathability of SCFa-PCs: (**I**) SCFa-PCs fabricated by coating methods, (**II**) SCY-PC-woven fabric, and (**III**) air permeability and moisture permeability data of PET fabric, SCY-PC-woven fabric, and SCFa-PCs fabricated by coating methods; (**f**) structural stability test of SCY-PC-woven fabric.

Based on the assembly mechanism of SCY-PCs, a prototype device for their continuous preparation was designed. As shown in Figure 5a, the continuous preparation process mainly consists of three stages: surface modification of polyester yarns, application of the assembly liquid, and thermal assembly. During the assembly process, the assembly box serves to supply sufficient thermal energy to facilitate the regular arrangement of the photonic crystals. Owing to its quasi-sealed configuration, the assembly box effectively minimizes heat dissipation, thereby maintaining a stable thermal environment for the self-assembly process. As shown in Figure 5(b(I)), the prepared SCY-PCs display bright and vivid structural colors, and the SCFa-PCs can be fabricated from SCY-PCs using weaving, embroidery, and knitting methods (Figure 5(b(II–IV))). Figure 5c shows the iridescent effect of SCY-PC-derived embroidered fabric. It can be observed that the SCY-PC-derived embroidered fabric possesses obvious angle dependence. As the viewing angle is increased, the structural color undergoes a transition from orange to green (Figure 5c, accompanied by a blueshift of the reflectance spectrum). Meanwhile, the breaking strength of SCY-PCs is higher than that of the original yarns, and the breaking elongation is also significantly increased (Figure 5d). This enhancement might stem from the presence of a PC layer assembled from hard-core/soft-shell nanospheres on the yarn surface. The PS core could provide high strength and rigidity, while the P(MMA-BA) soft shell may offer elasticity and flexibility. Under tensile force, the PC layer possibly suppresses local stress concentration, enabling the yarn to distribute external forces more effectively. Thus, both breaking strength and elongation are improved.

Figure 5e presents a comparative evaluation of the air permeability and moisture permeability between the SCY-PC-woven fabric and the SCFa-PCs fabricated by coating methods. As illustrated in Figure 5(e(I,II)), each fabric sample was placed over a beaker filled with hot water, and a transparent PET film was subsequently laid on top of each fabric. The PET film above the SCFa-PCs showed virtually no condensed water mist, whereas that above the SCY-PC-woven fabric exhibited pronounced water mist formation. This qualitative comparison indicates that the SCY-PC-woven fabric possesses superior air and moisture permeability relative to the SCFa-PC. Figure 5(e(III)) provides the quantitative measurements of air permeability and moisture permeability for the pristine PET fabric, the SCY-PC-woven fabric, and the SCFa-PCs. The data reveal that the air and moisture permeability of the SCY-PC-woven fabric (MP: 1762.178 g/m^2^·d, AR: 0.0858 Kpa·s/m) are comparable to those of the pristine PET fabric (MP: 1777.02 g/m^2^·d, AR: 0.07768 Kpa·s/m). In contrast, the SCFa-PCs exhibit a marked reduction in both air and moisture permeability (MP: 596.478 g/m^2^·d, AR: 271.6 Kpa·s/m). These results further corroborate that the SCY-PC-woven fabric not only displays brilliant structural colors but also retains excellent breathability and moisture transmission, highlighting its promising potential as a garment material.

As shown in Figure 5f, the SCY-PC-derived embroidered fabric was sequentially subjected to a series of durability tests, including immersion in acidic (pH = 3) and alkaline (pH = 11) solutions for 5 min each, 50 rubbing cycles, 50 brushing cycles, and 30 min of soap-washing. Following these treatments, no peeling or color fading was discernible by naked-eye observation. Moreover, the reflectance intensity remained essentially unchanged (Figure S7), and SEM images (Figure S8) revealed no observable damage to the PC structure, confirming the excellent structural stability of the SCY-PCs.

## 4. Conclusions

This work presents a robust strategy for the preparation of polyester yarns with high color fastness and high saturation structural coloration. PS@P(MMA-BA) hard-core/soft-shell colloidal nanospheres were employed as PC assembly units, while ionic surfactants were used to pre-treat and modify the polyester yarns. This modification significantly improves yarn wettability and, through electrostatic induction, promotes the rapid assembly and crystallization of colloidal nanospheres, generating bright and vivid structural colors and enabling the efficient preparation of SCY-PCs. Anionic and cationic surfactants demonstrate comparable electrostatic induction effects on the assembly of colloidal nanospheres, although the underlying electrostatic interactions differ in nature. According to the optical performance and fabrication considerations, an SDS concentration of 50 g/L was identified as the preferred anionic inducer. The SCY-PCs can be further processed into SCFa-PCs through weaving and embroidery, while maintaining vivid coloration and pronounced iridescent effects. Based on the ordered PC structure formed by the hard PS core and the elastic P(MMA-BA) soft shell, the SCFa-PCs show outstanding structural stability. No significant degradation in optical properties is observed after exposure to acidic and alkaline environments, brushing, rubbing, washing, or other mechanical stresses, demonstrating outstanding color fastness. In comparison with structurally colored fabrics prepared by conventional coating methods, SCFa-PCs derived from SCY-PCs show superior air and moisture permeability, indicating enhanced breathability and improved wearing performance. Therefore, this work offers a scalable strategy suitable for the industrial fabrication of structurally colored textiles.

## Data Availability

The original contributions presented in this study are included in the article. Further inquiries can be directed to the corresponding author.

## Supplementary Materials

The following supporting information can be downloaded at https://www.mdpi.com/article/10.3390/polym18192395/s1. Figure S1. Particle size distribution of PS@P(MMA-BA) nanospheres with different diameters. Figure S2. Contact angles of polyester yarns after treatment with varying dosages of SDS. Figure S3. Digital photographs of structurally colored fabrics modified by CTAB (a), SDS (b), and JFC (c), respectively. Figure S4. Effect of SDS dosage on the optical properties of SCY-PCs. (a–c) Digital photographs, optical microscope images, and reflectance spectra of the SCY-PCs, respectively. Figure S5. Optical microscope images of SCY-PCs prepared with different nanosphere mass fractions: (I) 13 wt%, (II) 25 wt%, (III) 30 wt%, (IV) 35 wt%, (V) 40 wt%, and (VI) 45 wt%. Figure S6. SEM images of SCY-PCs prepared with different nanosphere mass fractions: (I) 13 wt%, (II) 25 wt%, (III) 30 wt%, (IV) 35 wt%, (V) 40 wt%, and (VI) 45 wt%. Figure S7. Reflectance spectra of the SCY-PCs before and after the stability test. Figure S8. SEM images of SCY-PCs before (a) and after (b) the stability test.
