# Supplementary material for: Electrostatic Interaction-Induced Assembly of Colloidal Photonic Crystals on Polyester Yarns Applied for Structurally Colored Fabrics with High Breathability and Colorfastness
Source: Polymers (Basel). 2026 Sep 30;18(19):2395. doi: 10.3390/polym18192395 (PMC13666786; doi:10.3390/polym18192395)
Supplement: Supplementary file 1 [file polymers-18-02395-s001.zip › polymers-4595598-supplementary.pdf]

Electrostatic Interaction-Induced Assembly of Colloidal Photonic Crystals on Polyester Yarns Applied for Structurally Colored Fabrics with High Breathability and Colorfastness

[#]Xiaohui Wang[1,2], [#]Yalan Zhang[1], Xinyang Li[1], Jianjun Guo[1], Wanyang Sun[1], YiChen Li[1,3,4], Guojin Liu[1], Yi Huang[1], Minghua Wu[1], Jianzhong Shao[1,4] *

[1] *Engineering Research Centre for Eco-Dyeing and Finishing of Textiles, Ministry of Education, Zhejiang Sci-Tech University, Hangzhou 310018, China*

[2] *Rongsheng Petrochemical Co., LTD., Hangzhou 310018, China*

[3] *School of Textile and Clothing, Soochow University, Suzhou, Jiangsu 215127, China*

[4]*Hangzhou PhocryColor Sci-Tech Co., Ltd., Hangzhou, 311200, China*

[#] *These authors have contributed to this work equally.*

* *Corresponding author.*

E-mail address: jshao@zstu.edu.cn (J. Shao).

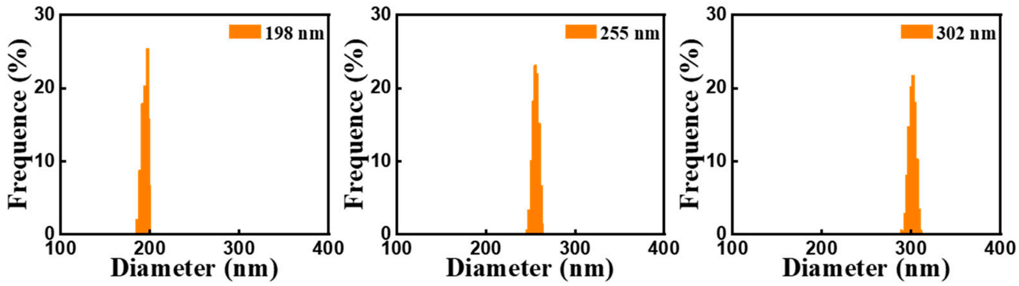

Figure S1 Particle size distribution of PS@P(MMA-BA) nanospheres with different diameters

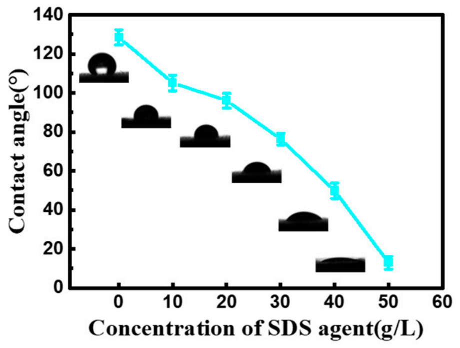

Figure S2 Contact angles of polyester yarns after treatment with varying dosages of SDS

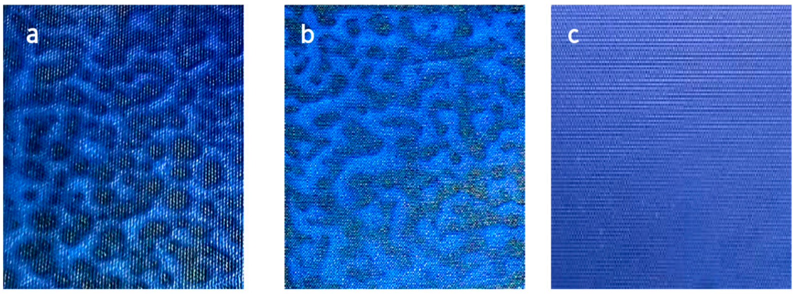

Figure S3 Digital photographs of structurally colored fabrics modified by CTAB (a), SDS (b), and JFC (c), respectively.

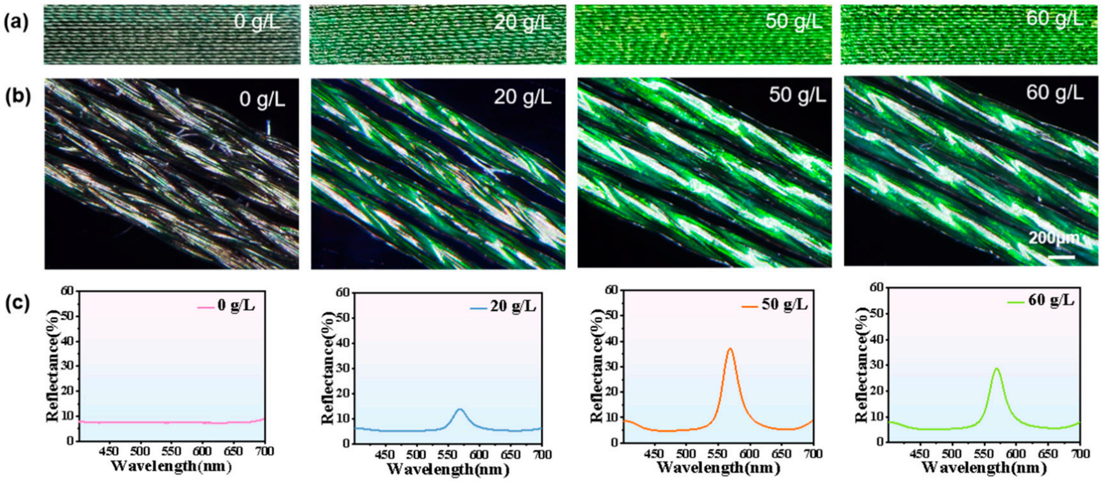

Figure S4 Effect of SDS dosage on the optical properties of SCY-PCs. (a–c) Digital photographs, optical microscope images, and reflectance spectra of the SCY-PCs, respectively.

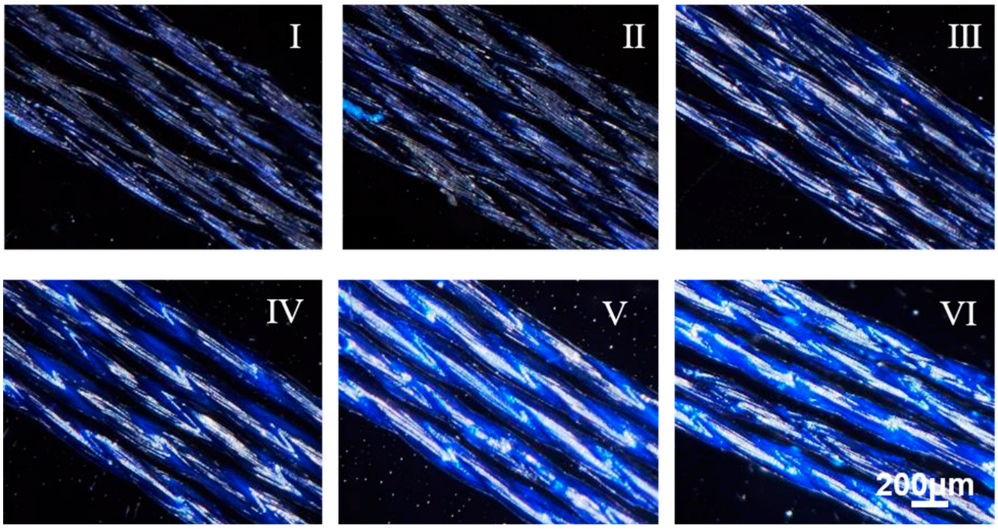

Figure S5 Optical microscope images of SCY-PCs prepared with different nanosphere mass fractions: (I) 13 wt%, (II) 25 wt%, (III) 30 wt%, (IV) 35 wt%, (V) 40 wt%, and (VI) 45 wt%.

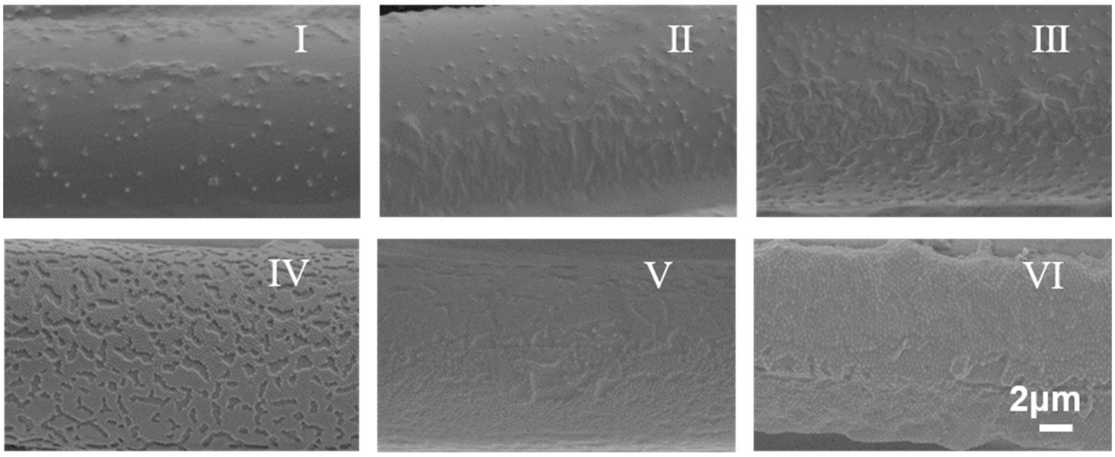

Figure S6 SEM images of SCY-PCs prepared with different nanosphere mass fractions: (I) 13 wt%, (II) 25 wt%, (III) 30 wt%, (IV) 35 wt%, (V) 40 wt%, and (VI) 45 wt%.

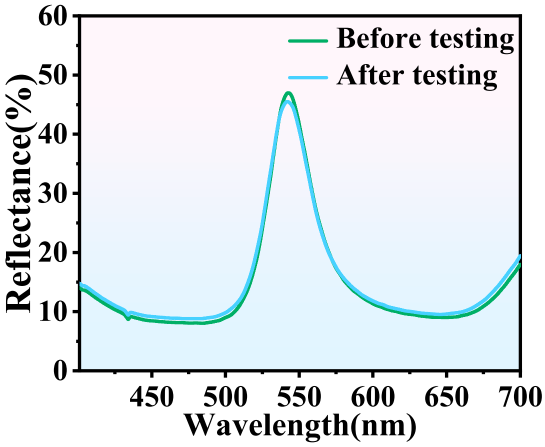

Figure S7 Reflectance spectra of the SCY-PCs before and after the stability test

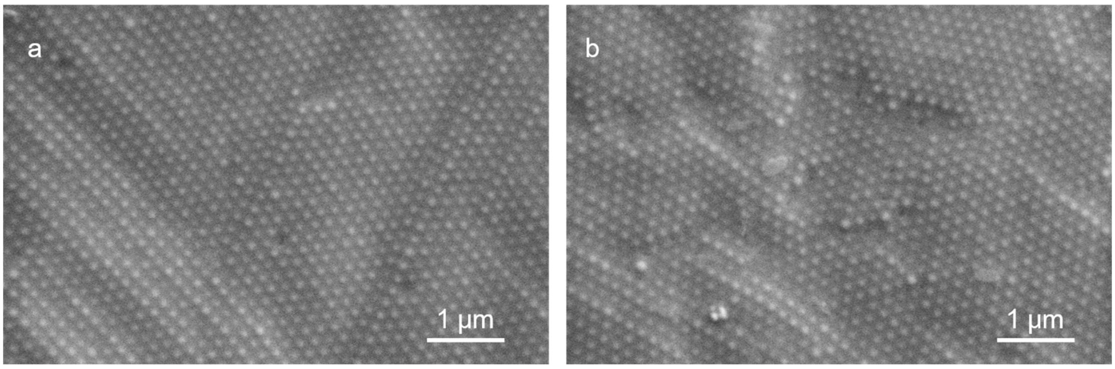

Figure S8 SEM images of SCY-PCs before (a) and after (b) the stability test
